# Oligonucleotide treatment causes flax β-glucanase up-regulation via changes in gene-body methylation

**DOI:** 10.1186/s12870-014-0261-z

**Published:** 2014-10-05

**Authors:** Wioleta Wojtasik, Anna Kulma, Aleksandra Boba, Jan Szopa

**Affiliations:** Faculty of Biotechnology, University of Wroclaw, Przybyszewskiego 63/77, Wroclaw, 51-148 Poland; Wroclaw Research Center EIT+, Stablowicka 147/149, Wroclaw, 54-066 Poland; Linum Foundation, Stablowicka 147/149, Wroclaw, 54-066 Poland

**Keywords:** Flax resistance, *Fusarium* infection, Epigenetically modified organism, DNA methylation/demethylation, OLIGO technology, β-1,3-glucanase

## Abstract

**Background:**

Nowadays, the challenge for biotechnology is to develop tools for agriculture and industry to provide plants characterized by productivity and quality that will satisfy the growing demand for different kinds of natural products. To meet the challenge, the generation and application of genetically modified plants is justified. However, the strong social resistance to genetically modified organisms and restrictive regulations in European Union countries necessitated the development of a new technology for new plant types generation which uses the knowledge resulting from analysis of genetically modified plants to generate favourably altered plants while omitting the introduction of heterologous genes to their genome. Four-year experiments led to the development of a technology inducing heritable epigenetic gene activation without transgenesis.

**Results:**

The method comprises the induction of changes in methylation/demethylation of the endogenous gene by the plant’s treatment with short oligodeoxynucleotides antisense to the coding region. *In vitro* cultured plants and F3 generation flax plants overproducing the β-1,3-glucanase gene (EMO-βGlu flax) were characterized by up-regulation of β-glucanase and chitinase genes, decreases in the methylation of CCGG sequences in the β-glucanase gene and in total DNA methylation and, more importantly, reasonable resistance against *Fusarium* infection. In addition, EMO-βGlu flax obtained by this technology showed similar features as those obtained by genetic engineering.

**Conclusion:**

To our best knowledge, this is the first report on plant gene activation by treatment with oligodeoxynucleotides homologous to the coding region of the gene. Apart from the evident effectiveness, the most important issue is that the EMO method allows generation of favourably altered plants, whose cultivation makes the plant producer independent from the complicated procedure of obtaining an agreement on GMO release into the environment and whose products might be more easily introduced to the global market.

**Electronic supplementary material:**

The online version of this article (doi:10.1186/s12870-014-0261-z) contains supplementary material, which is available to authorized users.

## Background

Flax (*Linum usitatissimum*) for centuries has played an important role in human society. This dual-purpose plant served as a source of fibres for manufacture of textile and oil for the chemical industry. The substantial increase in the availability of genomic and biological data helps researchers and breeders to utilize biotechnological approaches to improve the productivity and quality of flax products. For example, lignin content reduction by gene down-regulation resulted in an improvement of flax fibre quality [1–3]. Flax fibres modified by genetic engineering with polyhydroxybutyrate are used for composite materials in biomedical products such as implants and wound dressing [4]. Overproduction of different kinds of phenylpropanoid compounds in flax provides raw materials for such promising products as: oil with “ideal characteristics” which might serve as a dietary supplement protecting against atherosclerosis, seedcake extract constituting an “alternative antibiotic” effective in antibacterial protection, fibres successfully used for healing chronic ulcers, and micronized short fibres that appear to be suitable as a carrier and stabilizer of active substances (drugs, vitamins, hormones) [5–7]. Additionally, in all these cases improved plant resistance against *Fusarium* has been detected [8].

Beneficial properties of flax provide a strong argument for the renewal of its mass cultivation. In order to accelerate the interest in the cultivation of this crop, a constant concern for quality of seeds, and in particular a constant search for ways to improve the resistance of flax should be considered. It is estimated that about a 20% loss in the flax crop yield is caused by fungi. The diseases caused by them contribute to a reduction in the crop yield, seed and fibre quality, and therefore in the quality of products obtained from the crop, e.g. food, textiles, and animal feed. The most dangerous pathogens of flax are: *Fusarium oxysporum*, which penetrates flax cell roots and spreads throughout the plant via the vascular system, leading to root rot and wilt of leaves, and *Fusarium culmorum*, which causes rot at the base of the shoots, resulting in progressive plant necrosis and in consequence plant death [9,10].

The plant response to the pathogen infection may be local or systemic. The first reaction to the pathogen attack is a hypersensitivity reaction. It is manifested by the local necrosis of cells around the infected site and often causes changes in the metabolism of the plant leading to the production of metabolites of actuating mechanisms for inducing the systemic immunity. The immunity system is divided into two main types: systemic acquired resistance (SAR) and induced systemic resistance (ISR) [11].

In the systemic acquired response (SAR), comprising the tissue surrounding the site of infection and leading to a rise in the level of plant resistance to possible subsequent attacks by a pathogen, pathogenesis-related (PR) proteins are involved. They exhibit antifungal properties, associated with the enzymatic hydrolysis of chitin (PR-3, PR-8, PR-11) and β-glucans (PR-2), leading to the weakening and consequently to the destruction of the pathogen cell wall [12,13]. β-1,3-glucanases and chitinases act in at least two different ways: directly, by degrading the cell walls of the pathogen, and indirectly, by promoting the release of cell wall-derived materials that can act as elicitors of defence reactions, stimulating the production of other PR proteins and low molecular weight antifungal compounds, such as phytoalexins [14,15]. There is a correlation between expression of PR proteins in plants and their increased resistance to infections, for example in tomato resistant to *Alternaria solani* [16] or in potato resistant to *Phytophthora infestans* [17].

Currently, in order to increase the plant resistance to the pathogenic infection, genetic engineering is the most commonly used tool to produce genetically modified organisms (GMOs). Owing to targeted action this is the most effective method. The increase in the anti-pathogenic properties might be achieved by the overexpression of genes coding pathogenesis-related proteins and those involved in the synthesis of secondary metabolites [18–24].

Recently the new technology has been exploited for gene function studies in plants. The technology is based on plant treatment with high concentrations of a short (18-25 nucleotides), single stranded DNA fragment homologous to the targeted gene. This is a non-vector technology (OLIGO) of the gene modification and thus it is quite widely used in studies in mammalian cells, for example, to silence single genes in a cancer therapy [25]. This technique was applied for the first time in plant cells to induce changes in the expression of the gene encoding the transcription factor SUSIBA2 [26]. It was highlighted that the OLIGO technique might replace vector-mediated transformation (RNAi technology) because of the fact that both silence the gene with the same efficiency and produce a similar phenotype effect [27]. OLIGOs after being introduced into the plant cell may bind to a homologous sequence in a transcript and therefore activate RNA-dependent RNA polymerase (RdRP), which synthesizes a second strand, creating double-stranded RNA (dsRNA). This fragment can down-regulate the gene acting by an RNA interference (RNAi) mechanism [28].

Recently it has been shown that small exogenous DNA fragments might also activate homologous genes, if they are targeted to the regulatory sequences. Up to now, this new mechanism, termed RNA activation (RNAa), is ascribed only to mammalian cells. The RNAa mechanism appears to be Argonaute 2 dependent and is associated with histone changes (a loss of lysine-9 methylation on histone 3) at dsRNA-target sites [29,30]. The effects of the RNAa mechanism are shifted in time as compared to the RNAi mechanism (approximately 24-48 h), indicating different kinetics of both reactions. However, in plants the mechanism appears to be based on DNA methylation but not histone modifications. In addition, it has been shown that changes caused by the RNAa mechanism can be inherited after induction of epigenetic changes [31].

It is agreed that the molecular background for epigenetic changes is DNA methylation and, equally, a hypermethylation and a hypomethylation of the genome are often observed, and it does not matter what is the nature of inducing factors [32]. It seems that changes in the methylation of genes are one of the main epigenetic mechanisms of plant responses to stress conditions, or the introduction of foreign DNA into the genome. Double-stranded RNA fragments formed by the activity of RNA-dependent RNA polymerase or the activity of plant polymerase IV in the complex with polymerase V may recruit methyltransferase capable of DNA methylation and consequently the modification of gene expression [33]. Short double-stranded DNA fragments affect the methylation extent of regulatory fragments and/or coding portions of the homologous genes, and thus change their expression level. The process, which is mediated by an RNA-dependent DNA methylase (RdDM), can lead to either the activation or the repression of gene activity [34].

Recently, in order to improve the resistance of flax to the pathogen infection, we have generated a transgenic plant that overexpresses β-1,3-glucanase. The resulting transgenic flax (called type B) was characterized by a three-fold increase in resistance to *Fusarium oxysporum* and *Fusarium culmorum* infection. *In vitro*-cultured plants displayed a significant decrease in the content of carbohydrates, fatty acids and organic acids, and an increase in the levels of selected amino acids, polyamines and antioxidants [21]. In this work transgenic flax type B was used as a reference plant to new non-transgenic flax with induction of the endogenous β-1,3-glucanase gene.

The aim of this research was to develop the non-invasive technology for the generation of a new type of flax based on the epigenetic modulation of nucleic acids. In particular, the main goal was to produce a new, genetically stable flax with increased expression of endogenous genes encoding β-1,3-glucanase, which therefore results in improved resistance of flax to *Fusarium oxysporum* and *Fusarium culmorum* infection.

## Results

### Analysis of the expression of PR genes and PME1

**Figure 1:**
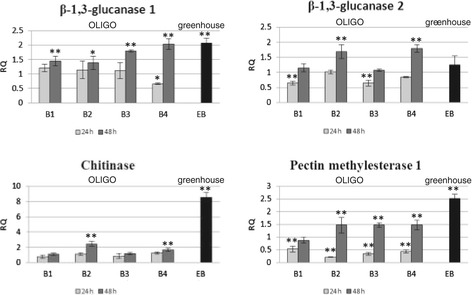
**Expression levels of PR genes and PME1 gene.** The mRNA level of β-1,3-glucanases, chitinase and pectin methylesterase 1 genes in flax treated with OLIGOs (B1, B2, B3 and B4) at 24 h (light grey bars) and 48 h (dark grey bars) after exposure to OLIGOs in comparison with control, non-treated flax and in EMO-βGlu flax (EB) (black bars) in comparison with control flax was obtained from the real-time RT-PCR analysis. Actin was used as a reference gene and the transcript levels were normalized to those of the control plant (C = 1; not presented in the figure). Data represent the mean ± standard deviations from three independent experiments. The significance of the differences between the means was determined using Student’s t test (*P < 0.05, **P < 0.01). RQ – relative quantity.

The mRNA levels of selected PR proteins (two isoforms of β-1,3-glucanase and one isoform of chitinase) and pectin methylesterase 1 (PME1), another protein participating in the plant response to stress, were determined in the flax treated with OLIGOs and in the F3 generation of the selected line labelled EMO-βGlu. The obtained results are presented in Figure 1.

The analysis of the β-1,3-glucanases revealed enhanced expression of the first isoform (up to 2-fold for B4) in flax treated with four OLIGOs and increased expression of the second isoform in flax treated with OLIGOs B2 and B4 (up to 1.7-fold and 1.8-fold, respectively) at 48 hours after exposure to OLIGOs. Moreover, OLIGOs B1 and B3 reduced the mRNA levels of the second isoform of β-1,3-glucanase at 24 hours (by 65%) but did not affect its expression at 48 hours.

Concomitantly, the activation of two other genes that commonly respond to environmental stress, chitinase and pectin methylesterase 1, were detected. The mRNA level of the pectin methylesterase 1 gene in flax treated with OLIGOs at 24 hours after exposure to OLIGOs was reduced (53%, 22%, 35% and 44% for B1, B2, B3 and B4, respectively) in comparison with the control, while at 48 hours OLIGOs B2, B3 and B4 caused up to 1.5-fold higher expression. The gene encoding chitinase was found to be activated after 48 hours by B2 (2.45-fold) and B4 (1.7-fold).

Similarly to the flax treated with OLIGOs, the expression of genes – the first isoform of β-1,3-glucanase, chitinase and pectin methylesterase 1 in F3 generation plants – was significantly higher than in the control. The transcript level of β-1,3-glucanase, chitinase and PME1 was 2-fold, 8.5-fold and 2.5-fold higher, respectively, in the EMO-βGlu plants. Changes in the expression of PR genes involved in SAR were related to the increase of plant resistance to the pathogen infection.

### Callose content

**Figure 2:**
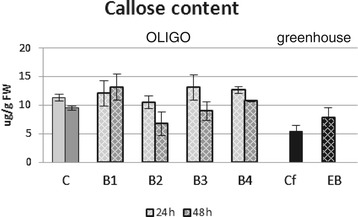
**Callose content.** Analyses of flax treated with OLIGOs (B1, B2, B3 and B4) at 24 h (light grey bars) and 48 h (dark black bars) after exposure to OLIGOs in comparison with control flax (C) and EMO-βGlu flax (EB) in comparison with control flax (Cf) were performed as described in the experimental procedure section. Data represent the mean values ± SD from five independent experiments.

The induction of activity of β-glucanase genes was not reflected in the respective enzyme activity measured as the callose content. Figure 2 shows that the callose content decreased only in flax treated with two OLIGOs, B2 and B3, but these changes were not statistically significant. In the rest of the samples, the level of callose was similar to the control.

In contrast, a 1.5-fold increase in the callose content was observed in the EMO-βGlu plants. The increase in the callose content in F3 generation plants may be due to the increased expression of one of the genes controlling the process of callose synthesis (Additional file 1: Figure S2P), where a 1.6-fold increase in the expression of the CALS2 gene was detected.

### Pattern of DNA methylation of β-1,3-glucanase gene

The determination of DNA methylation of the β-1,3-glucanase gene in the flax treated with OLIGOs and in the EMO-βGlu plants was made in three promoter sites, in three exon sites and in one intron site.

**Figure 3:**
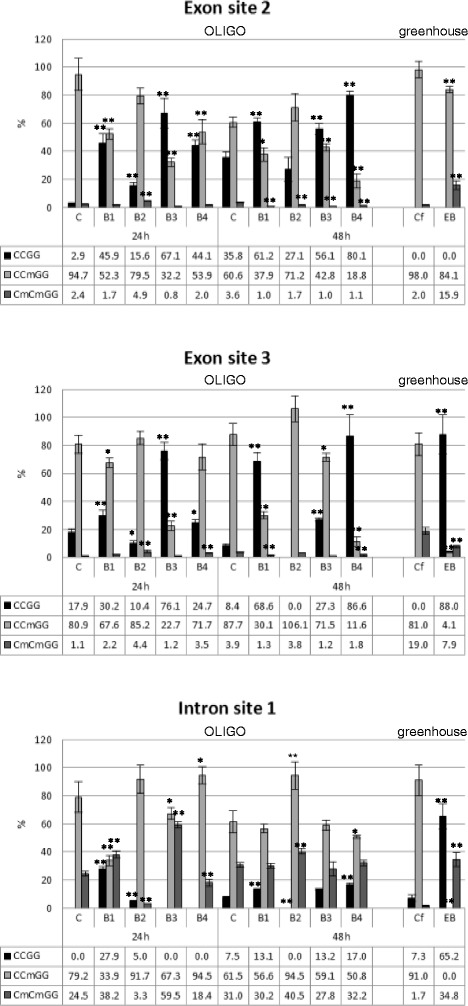
**Methylation of crucial sites of β-1,3-glucanase gene.** The analysis of the methylation of two sites in exons and one site in the intron of flax treated with OLIGOs (B1, B2, B3 and B4) at 24 h and 48 h after exposure to OLIGOs in comparison with control, non-treated flax (C) and in EMO-βGlu flax (EB) in comparison with control flax from field (Cf) was determined by digesting genomic DNA with restriction enzymes HpaII-MspI with subsequent semi-quantitative PCR reaction, the separation of PCR product on agarose gel and the quantification of the bands by densitometry. Data represent the mean ± standard deviations from three independent experiments. The significance of the differences between the means was determined using Student’s t test (*P < 0.05, **P < 0.01).

The results obtained from the study of the DNA methylation of the β-1,3-glucanase gene in flax treated with OLIGOs and in the EMO-βGlu plants showed a similar profile of the methylation for all checked sites. Three analysed promoter sites and one site in the exon remained unchanged (Additional file 2: Figure S1). However, the second and the third site in exons were in most cases demethylated at the internal cytosine of the CCGG sequence. Also the analysed first site in the intron was demethylated in plants treated with OLIGOs (Figure 3). The demethylation of the second exon site did not change substantially between particular plants treated with selected OLIGOs B1, B3 and B4 at either 24 or 48 hours after exposure to OLIGOs. However, a smaller change and a lack of changes in the demethylation of the internal cytosine of the CCGG sequences were observed at 24 and 48 hours after B2 treatment. In the third exon site of β-1,3-glucanase the highest demethylation occurred in flax treated with OLIGO B3 after 24 hours and in flax treated with B1 and B4 after 48 hours. Interesting changes of DNA methylation were observed in the intron, where after 24 hours only OLIGO B1 caused a reduced level of cytosine methylation. Moreover, 48 hours after plants’ treatment with OLIGOs, B1, B3 and B4 had a similar pattern of methylation, with 13-17% of CCGG sites unmethylated.

Similarly to the plants after exposure to OLIGOs, F3 generation plants called EMO-βGlu showed increased levels of demethylation in the third position in the exon and the first site in the intron. However, in these plants methylation of the second site in the exon was observed. Similarly, the other potential methylation sites (three sites in the promoter and one site in the exon) were not changed (Additional file 2: Figure S1).

### Analysis of expression of genes involved in the OLIGO mechanism

**Figure 4:**
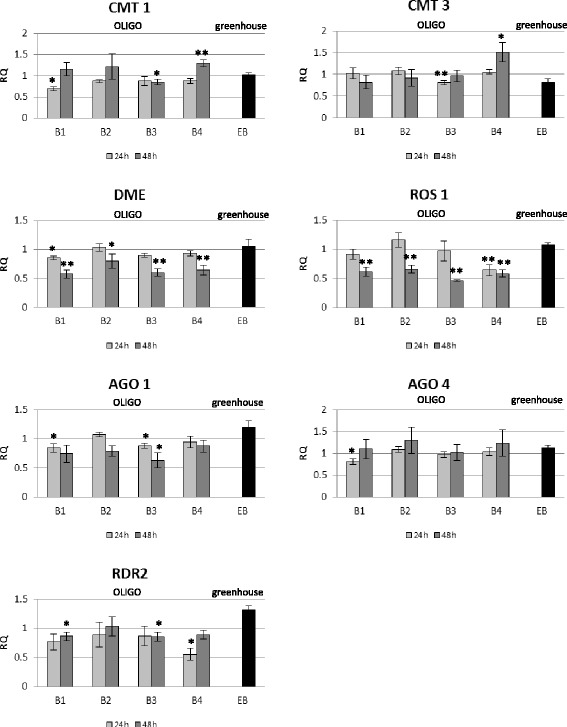
**Expression level of genes involved in the OLIGO mechanism.** The mRNA level of CMT1 (chromomethylase 1), CMT3 (chromomethylase 3), DME (Demeter), ROS1 (repressor of silencing 1), AGO1 (Argonaute 1), AGO4 (Argonaute 4), RDR2 (RNA-depended RNA polymerase 2) in flax treated with OLIGOs (B1, B2, B3 and B4) at 24 h (light grey bars) and 48 h (dark grey bars) after exposure to OLIGOs in comparison with control, non-treated flax and in EMO-βGlu flax (EB) (black bars) in comparison with control flax was obtained from the real-time RT-PCR analysis. Actin was used as a reference gene and the transcript levels were normalized to those of the control plant (C = 1; not presented in the figure). Data represent the mean ± standard deviations from three independent experiments. The significance of the differences between the means was determined using Student’s t test (*P < 0.05, **P < 0.01). RQ – relative quantity.

To explain the possible mechanism of OLIGO action, the expression levels of genes involved in the RNAi and RNAa mechanism – RNA-dependent RNA polymerase, AGO1 and AGO4 – were investigated. Assuming that the changes could also refer to the methylase/demethylase genes, expression levels of genes encoding methylases (CMT1, CMT3) and demethylases (ROS1, DME) were measured (Figure 4).

Flax plants at 24 hours after exposure to OLIGOs were characterized by minor changes in the expression of the tested genes. OLIGO 1 (B1) induced the reduction of the transcript level of the CMT1 gene (to 70%), DME gene (to 85%), AGO1 (to 85%) and AGO4 (to 76%). Also OLIGO 3 (B3) caused a decrease in expression of the CMT1 gene (to 84%), CMT3 (to 80%) and AGO1 (to 87%), whereas OLIGO 2 and 4 had no effect on expression levels of investigated genes.

Flax plants at 48 hours after exposure to OLIGO 4 showed increased expression of the CMT1 gene (up to 1.3-fold) and CMT3 gene (up to 1.5-fold). The mRNA levels of both demethylases (DME and ROS1) were reduced to 80-50% by the action of all OLIGOs. Also expression of the RDR2 gene was only slightly changed in flax treated with OLIGOs (especially B1 and B3).

In the F3 generation flax plants called EMO-βGlu the expression levels of methylases and demethylases were similar to the control. Only a slightly increased level of mRNA was observed for AGO1 and RDR2 genes.

### Total level of DNA methylation

**Figure 5:**
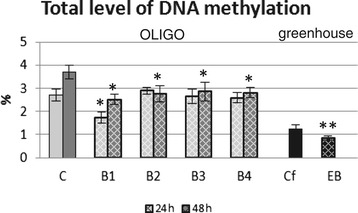
**Total level of DNA methylation.** Analyses of flax treated with OLIGOs (B1, B2, B3 and B4) at 24 h (light grey bars) and 48 h (dark black bars) after exposure to OLIGOs in comparison with control flax (C) and EMO-βGlu flax (EB) in comparison with control flax (Cf) were performed as described in the experimental procedure section. Data represent the mean values ± SD from three independent experiments (*P < 0.05).

As a result of the exposure of flax to oligonucleotides the changes in the total level of the genomic DNA methylation were observed and presented in Figure 5. After 24 hours only B1 oligonucleotides caused a decrease (by 35%) in the DNA methylation level, while after 48 hours all used oligonucleotides affected the methylation status of genomic DNA (25% decrease).

Also, the total level of DNA methylation in EMO-βGlu plants was determined (Figure 5), and it was lower in EMO-βGlu plants as compared to control plants. Such a change, in accordance with the principles of epigenetics, may cause activation of genes.

### Secondary structure of mRNA and OLIGOs and correlation between secondary structure of OLIGOs and expression level of β-1,3-glucanase gene

**Figure 6:**
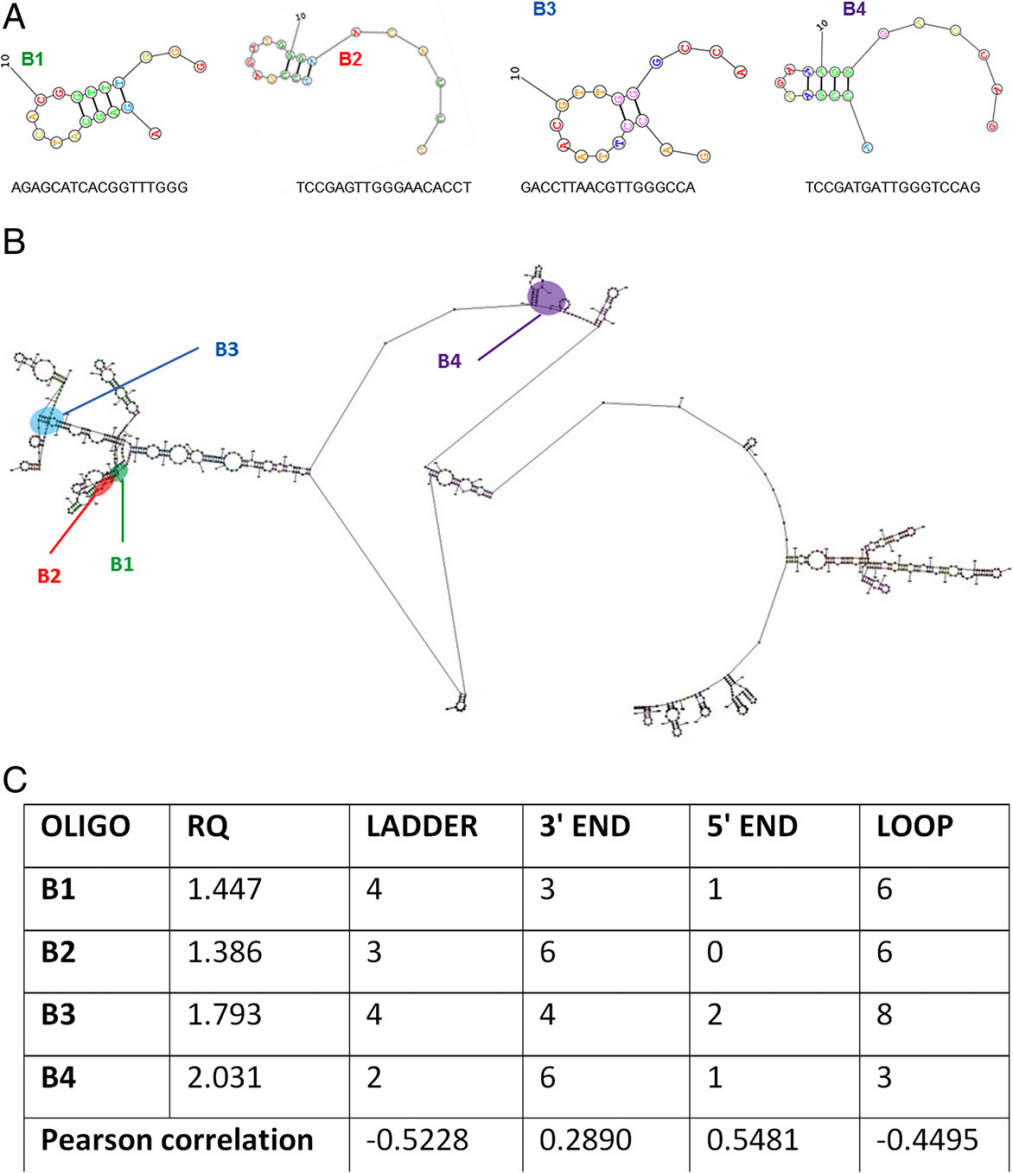
**Predicted secondary structure of OLIGOs and β-1,3-glucanase mRNA and correlation coefficients between secondary structure of OLIGOs and expression level of β-1,3-glucanase gene. (A)** Nucleotide sequences of OLIGOs (B1, B2, B3 and B4) and their predicted secondary structures. **(B)** The predicted secondary structure of β-1,3-glucanase mRNA and the binding sites of OLIGOs (B1, B2, B3 and B4). **(C)** Correlation coefficients between secondary structure of OLIGOs and expression level of β-1,3-glucanase gene.

Among several designed and tested OLIGOs the four most effective in gene (β-glucanase) activation were chosen for further analysis. The nucleotide sequences of selected OLIGOs (B1, B2, B3 and B4) and their predicted secondary structures were presented in Figure 6A. The predicted secondary structure of mRNA from the β-1,3-glucanase gene and OLIGO binding sites were shown in Figure 6B.

Both the OLIGOs (B1, B2, B3 and B4) and mRNA were characterized by a great diversity of secondary structures in the sense that three main types could be recognized: a stem (paired, double-stranded fragments), a single-stranded loop, and a single-stranded chain whose length was variable. The mutual location of OLIGO complementary to the mRNA sequence was very close (B1 and B2), of average distance (B3) and very distant (B4) to each other.

The obtained data were used to calculate the correlation of identified parameters such as the secondary OLIGOs/mRNA structure and the methylation pattern with expression of the β-1,3-glucanase gene. The highest level of correlation was observed for the length of the single-stranded 5'-terminus and the length of the paired sequence fragment of OLIGOs (Figure 6C). Thus, the secondary structure of OLIGO was important for its effect on homologous sequences and the activity of the endogenous gene. The most important information was obtained from the analysis of the methylation profile of the endogenous gene under the influence of OLIGOs. Whereas in all promoter methylation sites, in the intron site and in the first exon site the changes in the methylation pattern were non-existent, the third site in the exon in most cases was demethylated in plants treated with OLIGOs. Thus, it might suggest that the β-glucanase gene activation upon OLIGO treatment is probably due to demethylation of the gene coding region.

### Resistance of EMO-βGlu plants to *Fusarium* infection

**Figure 7:**
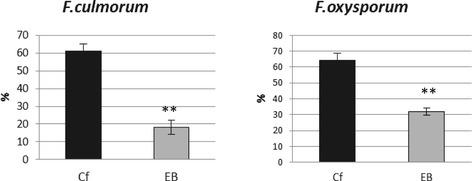
**Resistance of EMO-βGlu flax and control flax to fungal infection.** The resistance of EMO-βGlu flax (EB) in comparison with control flax (Cf) to *Fusarium culmorum* and *Fusarium oxysporum* was determined as % of infected flax seedlings. Data represent the mean values ± SD from three independent experiments (**P < 0.01).

The consequence of activation of the gene response to the infection in flax was an increase in the plant resistance to the *Fusarium culmorum* and *Fusarium oxysporum* infection, as shown in Figure 7. Obtained data indicated that more than threefold increase in resistance of flax EMO-βGlu to the *F. culmorum* infection and more than twice to the *F.oxysporum* infection were observed. The resistance of EMO-βGlu plants was even higher than those ascribed to transgenic B-type plants [21].

### Analysis of the main polymers in the cell wall of EMO-βGlu plants

Important components of the plant defence against pathogen infection are the cell wall polymers. We evaluated the expression of genes involved in the synthesis and degradation of pectin (A and B), the level of pectin (C and D), the expression of genes participating in the synthesis and degradation of hemicellulose (E and F), the level of hemicellulose (G and H), the cellulose metabolism genes (I), lignin genes (K) and the level of these polymers (J and L), the level of mRNA of genes involved in polyamine synthesis and degradation (M and N), the level of these compounds (O) and the expression of callose synthesis genes (P) in EMO-βGlu plants (Additional file 1: Figure S2A-P). The vast majority of genes involved in pectin, hemicellulose, lignin and cellulose metabolism were activated in EMO-βGlu plants, while the metabolic products of their activity only in certain cases reflected these activations.

Minor changes in the total level of pectic and hemicellulose sugars were observed (a slight increase in NSF and K1SF fractions); however, significant changes occurred in the uronic acid content of individual fractions of both polymers. Thus, the reorganization of pectin and hemicellulose polymers rather than changes of their amount were the response to the change in the activity of β-glucanase genes and genes of these polymers’ metabolism. Similarly, an insignificant change in the level of lignin in EMO-βGlu plants was observed, although more than 3-fold activation of certain genes (such as SAD – sinapyl alcohol dehydrogenase) was observed. The biggest changes were measured for the cellulose level, which corresponds to an increase in the gene expression of this polymer’s metabolism (up to 1.6-fold for CSL1; cellulose synthase 1 and up to 1.7-fold for CSL4; cellulose synthase 4) in EMO-βGlu plants. Also, the level of polyamines was elevated (2-fold); however, this change could not be easily correlated with the expression of polyamine metabolism genes.

## Discussion

GMO technology is the most exploited method in modern plant biotechnology for generation of transgenic organisms for both scientific and commercial purposes. However, according to Eurobarometer over 60% (61-90% depending on the country) of society does not accept GMO, which locks the door for the commercialization of GM plants and products. Furthermore, the GM technology is laborious and time-consuming. Thus, the challenge for new biotechnology is to develop a method of modulation rather than modification of the genome which, however, is similar to the agrotransformation effect.

The new technique of non-invasive modulation of the genome exploited in this work is based on the induction of DNA modification. Since the new technology does not use non-linear nucleotide sequences and the way of introducing them into the plant is the vascular system, the technology can be regarded as natural, and therefore not involving genetic modification. The obtained plants overproducing β-glucanase are defined as epigenetically modified organisms (EMO) and they are treated as an alternative to GMO.

The fundamental importance of the β-glucanase gene in flax resistance to pathogen infection was identified by in-depth investigation of previously generated transgenic plants overexpressing the heterologous (potato) β-glucanase gene [21]. Besides the improved resistance against the pathogen infection, the characteristic feature of these plants was changes in carbohydrate, phenolic and polyamine contents. The interesting finding was that despite the transgene (β-glucanase) high expression, modified flax showed a large increase in the endogenous chitinase gene. There are several reports describing the induction of plant resistance to pathogen infection upon the overexpression of either β-glucanase or chitinase or both simultaneously [35,36]. However, none of these studies report transgene-endogenous gene cross-activation. We speculate that enzyme released upon overproduction (derived from the β-glucanase transgene) affect the cell wall compounds and induce endogenous counterparts. Indeed, the product of β-glucanase might operate as an elicitor activating plant metabolism. For example, linear β-1,3 glucans are elicitors of defence responses in tobacco, which activate phenylalanine ammonia-lyase, caffeic acid O-methyltransferase and lipoxygenase [37].

The increased plant resistance to *Fusarium* concomitantly with favourable changes in flax metabolism was a sufficient reason for the development of epigenetically modified plants and thus strengthening the market potential of flax.

For many years the epigenetic modification of mammalian cells has been successfully exploited. Although successful, the molecular mechanism behind this new technology of gene activity modulation is as yet not fully understood. Considering the data already published, it is assumed that these mechanisms might include gene silencing called RNA interference (RNAi) or gene activation by mRNA biosynthesis (RNAa) or modification of gene expression by DNA methylation of cytosines and/or histones.

In the case of plants, there are only a few reports that have evidenced the down-regulatory action of oligodeoxynucleotides (OLIGOs) on homologous plant genes. For example, antisense OLIGOs were used to reduce the expression of the nucleus-encoded phytoene desaturase in tobacco, chlorophyll a/b-binding genes in wheat, the chloroplast-encoded psbA gene in *Arabidopsis thaliana* [38], SUSIBA2, a transcription factor, in sugar-treated barley [26] and the GNOM LIKE 1 gene in tobacco [27]. In all these cases, plant treatment resulted in gene down-regulation, probably by an RNAi mechanism. There is however no report on the up-regulatory role of OLIGOs in plants, which is in contrast to mammalian cells.

In order to exploit this new technology for flax improvement, more than 70 different OLIGOs, 18 nucleotides long, were investigated for their influence on homologous genes from the flax terpenoid pathway. A study of 9 different genes from this pathway revealed that 40% of them were down-regulated, 46% were not affected and, most importantly, 14% of them were up-regulated (data not yet published).

Thus, we took the opportunity to treat the flax plants with several generated OLIGOs homologous to different regions in the β-glucanase gene. Successfully, four of them showed endogenous gene activation and plants activated in this way were further analysed. *In vitro* cultured plants showed an increase in β-glucanase gene expression accompanied by demethylation of CCGG sequences in it. This is in agreement with the general view that gene demethylation results in its higher activity [39–41]. The detailed analyses of cultured plants provide data on changed activity of several other genes involved in both primary and secondary metabolism including chitinase, pectin methylesterase 1 and those engaged in cell wall metabolism. The reason for such global metabolism changes is as yet unknown. We speculate that overproduction of β-glucanase might lead to accumulation of elicitors that elicit plant metabolism by for example genome demethylation. Indeed, partial demethylation of total genomic DNA was detected, which supports this speculation. Also the expression analysis of genes involved in the process of DNA methylation partially reflects the reduction in DNA methylation level. For example, demethylase genes (DME, ROS1) are strongly down-regulated in all cases. However, in most cases methylase genes are also down-regulated. Perhaps the final DNA methylation status is regulated by the interplay between methylase and demethylase genes.

Although the molecular mechanism behind plant treatment with OLIGOs is not fully understood, it is clear from this report that they might activate homologous gene expression and this event is accompanied by gene demethylation. This is corroborated by several reports concluding that genes are regulated by methylation/demethylation status [42–44].

The importance of crop plant gene modulation using EMO technology might be limited by the stability over plant generations. In order to study the stability of flax features induced by OLIGOs, third generation (F3) plants called EMO-βGlu were investigated. Similarly to the F0 generation and reference transgenic B-type plants, these EMO-βGlu type F3 plants were characterized by β-glucanase and chitinase gene up-regulation and demethylation of CCGG sequences in the β-glucanase gene. Changes in primary and secondary plant metabolism concomitant with the decrease in total DNA methylation were also detected. The process involved alteration in methylase (CMT3 – down-regulated) and demethylases (ROS1, DME – up-regulated) gene expression. The importance of gene-body methylation for gene expression is still not well understood, but in most cases highly expressed genes have low levels of methylation. Since several different classes of gene expression were changed upon OLIGO treatment, the mechanism behind it is presumably of general nature. Thus it is speculated that OLIGO treatment induces genome rearrangement and decreases in total DNA methylation, and 48 h plant exposure is sufficient to do this and to keep this rearrangement inherited. Perhaps the direct signal for genome rearrangement derives from the action of the activated β-glucanase gene resulting in production of elicitors. This view is supported by very similar molecular characteristics of EMO-βGlu and reference GM flax.

Many literature reports have described changes in the methylation pattern of genes resulting from exposure to physical and chemical treatment. For example, *Arabidopsis* plants subjected to low temperature stress [45] showed extensive changes in methylation of the genomic DNA of these plants. The induction of epigenetic changes in DNA methylation of the genome was achieved by treating plants with jasmonic acid (JA) and salicylic acid (SA). An experiment with *Taraxacum officinale* showed not only extensive methylation but also that 74% to 92% of the variation in the level of methylation induced by JA and SA, which was preserved in the next generation [46].

The importance of changes in expression of flax genes and metabolism upon OLIGOs treatment for plant resistance against *Fusarium* infection was investigated. Increased expression of PR2 (β-glucanase and chitinase) family genes concomitant with the accumulation of polymers (lignin, cellulose) and polyamines is perhaps the molecular background behind it. Both enzymes are well known to be involved in the plant response to pathogen infection. Cell wall polymers are also active in cell protection and polyamines are signal molecules in this response.

Since EMO-βGlu plants were similar to the plants treated with OLIGOs in terms of changes in the expression of β-glucanases and the profile of β-glucanase gene methylation, and, most importantly, similar to stable transgenic B-type plants in terms of resistance to infection, it might be suggested that the changes caused by OLIGOs were inherited. Therefore, this new type of plant was called EMO-βGlu (from epigenetically modified organisms of β-glucanase), and it is a remarkable alternative to GM plants.

**Figure 8:**
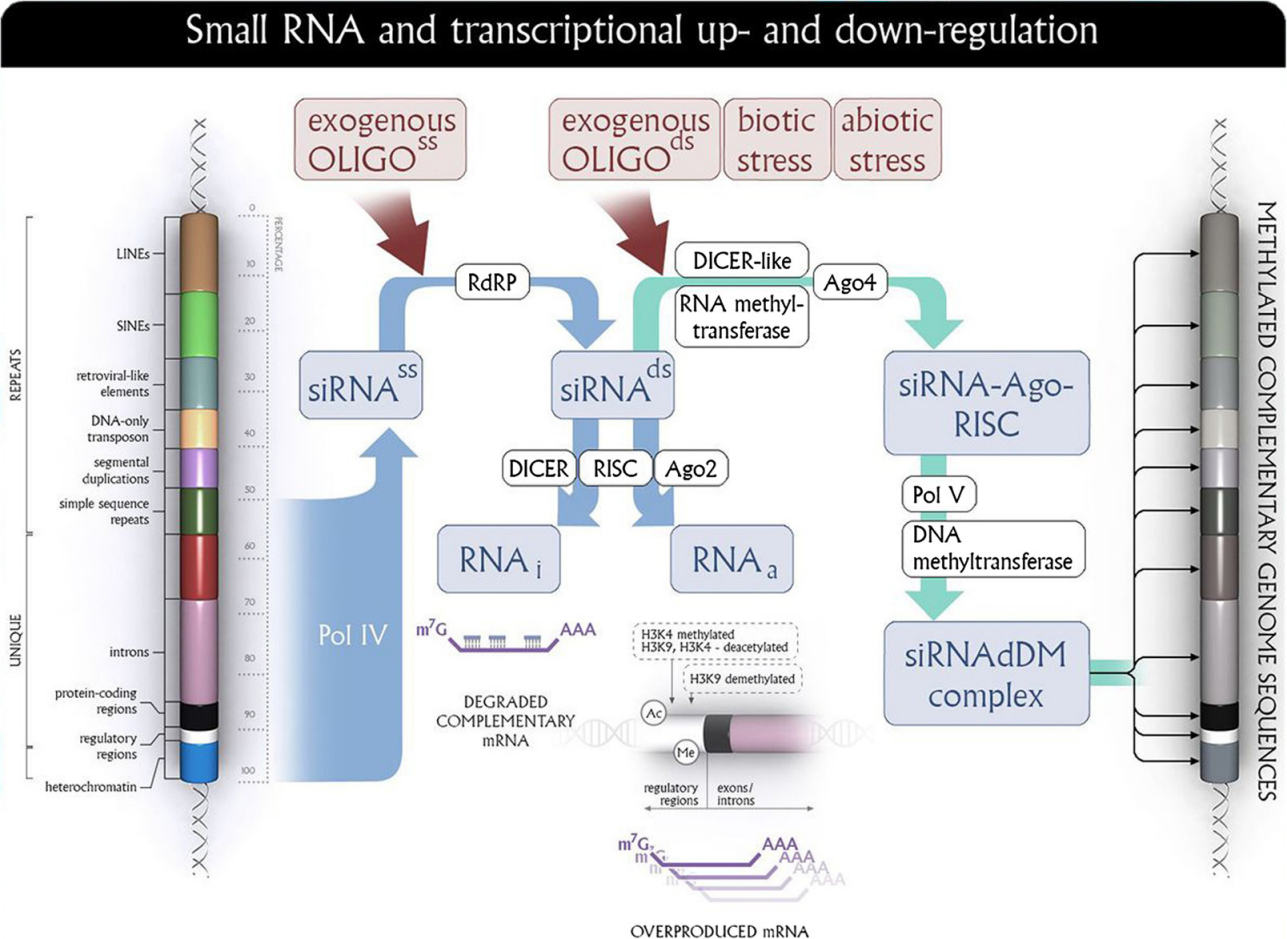
**Effect of oligonucleotides on the metabolism of the homologous mRNA or the modulation of homologous DNA.** A short (18-25 nucleotides), single stranded DNA fragment when introduced into a plant cell may bind to a homologues sequence in a transcript and therefore activate RNA-dependent RNA polymerase (RdRP). RdRP synthesizes a second strand creating double-stranded RNA (dsRNA), whose presence brings to action RISC and DICER complexes, and Argonaute (AGO) proteins. The dsRNA fragment could down-regulate the gene by an RNA interference (RNAi) mechanism and/or may also activate the target gene by an RNA activatory (RNAa) mechanism. Furthermore, the presence of dsRNA can also provoke RNA dependent methylation of the homologous DNA fragment. Involved in this process are AGO proteins, DNA methyltransferases, demethylases, and, present only in plants, polymerase IV and V.

EMO technology is based on the use of short oligonucleotide fragments (OLIGOs) introduced into plants in a non-invasive way. The use of short, single-stranded DNA fragments allows one to alter the expression of homologous genes. The efficiency of the technology depends on the secondary structure of OLIGO used. The highest level of correlation for OLIGOs structure and the expression of the β-1,3-glucanase gene was observed for the length of the single-stranded 5'-terminus and the length of the paired sequence fragment of OLIGOs. The technique does not require the use of a vector and consists of the infiltration or the uptake with nutrient (sucrose) of short, 12-21 nt single-stranded DNA sequences (OLIGOs) in the sense or antisense orientation complementary to the coding and/or regulatory sequences of the modulated gene. It is assumed that subsequently produced (endogenous) duplex RNAi and/or RNAa and/or siRNAdDM (RNAi associated with methyltransferase) as a product of activity of respective endogenous, AGO-dependent enzymes (RNA-dependent RNA polymerase, DICER and RISC complexes, DNA methyltransferase) become a signal for gene expression regulation. These mechanisms include gene silencing called RNA interference (RNAi) and/or gene activation by mRNA biosynthesis (RNAa) and/or modification of gene expression by DNA methylation of cytosines. All three potential mechanisms are shown in Figure 8. So far, the mechanism of RNAi is the best studied, while the other two require further investigation.

## Conclusion

In summary, the data accumulated suggest that the OLIGO technology is relatively very simple and effective enough to achieve improved flax, modified in the preferred direction and stable in at least up to the third generation. The non-transgenic plants with the modulated genome showed the same characteristics as the reference transgenic plants. Thus, it provides a new tool for crop plant improvement based on the epigenetic modulation of nucleic acids.

The authors of this paper inform that the EMO-βGlu flax seeds are available for scientific purposes upon request.

## Methods

### Plant material

Flax seeds (*Linum usitatissimum* L., cv. *Nike*) were obtained from the Flax and Hemp Collection of the Institute of Natural Fibres in Poland. Seed germination was carried out on Murashige and Skoog medium (Sigma-Aldrich) solidified with 0.8% agar and supplemented with 1% sucrose at a 16 h light (21°C), 8 h darkness (16°C) regime on Petri dishes.

### Designing OLIGO complementary to different gene part

OLIGOs were designed using the MFOLD software (Genetics Computer Group, Madison), which provides the best RNA binding sites, and also takes into account the possibility of secondary structure folding that may facilitate or impede binding to sequences. While designing OLIGO, we took into consideration the nucleotide composition, the melting temperature and the number of GC pairs, which are crucial in annealing OLIGO to the homologous gene sequences.

The 4 OLIGOs (B1, B2, B3 and B4) in the antisense orientation were designed for exon sites of β-1,3-glucanase (Acc. No. JQ670874) and were not complementary to mRNA of other isoforms of β-1,3-glucanase from the flax genome. All of them were characterized by the following physical constants: length – 18 nucleotides; C + G – 55.6%; Tm – 50.3°C. They had the following sequences and positions (starting from ATG sequence):

B1 - 3’AGAGCATCACGGTTTGGG 5’; DNA: 220-237; mRNA: 129-146.

B2 - 3’TCCGAGTTGGGAACACCT 5’; DNA: 274-291; mRNA: 183-200.

B3 - 3’GACCTTAACGTTGGGCCA 5’; DNA: 356-373: mRNA: 264-281.

B4 - 3’TCCGATGATTGGGTCCAG 5’; DNA: 1501-1518; mRNA: 493-510.

The sequences of B1 and B2 were located very close together, the B3 sequence was near to B1 and B2, and the B4 sequence was very distant from the others in the structure of mRNA β-1,3-glucanase. OLIGOs were synthesized by Genomed S.A. (Poland).

### Plant treatment with OLIGO

To introduce OLIGOs into the flax, 4-week-old plants grown in the tissue culture were transferred into the 10 μM OLIGO solution in water and then infiltration for 15 minutes in a vacuum was carried out. Approximately 20 plants were used for each oligodeoxynucleotide treatment. Next, flax plants were transferred onto MS medium and after 24 and 48 hours a tissue sample from each plant was collected for further analysis. After one week the plants were transferred into soil and grown in the greenhouse until maturation. The collected seeds were labelled F1.

The F0 plants were pre-selected based on β-glucanase expression. Approximately 15 plants from each OLIGO treatment showed an increase in β-glucanase gene expression. The DNA methylation and detailed gene expression analysis was done on 5 plants showing the highest increase in β-glucanase gene expression. Seeds from those plants were collected and then grown in the greenhouse, under optimized growth conditions, until obtaining matured seeds of the F2 generation. The F1 and F2 generation plants were analysed like F0 plants in order to check the presence of the desirable trait (not shown) and after each generation the best lines were selected for further cultivation. From the F2 generation one of the lines derived from B4 OLIGO was selected as the most stable one and with highest expression of the β-glucanase gene. Harvested and pooled seeds of this plant (F2 generation) were again grown in the greenhouse and green part samples of these plants, called EMO-βGlu (F3 generation), were analysed throughout this work.

### Gene expression analysis

The mRNA level for the investigated genes was determined using real-time PCR. The total RNA was isolated using the Trizol method (Invitrogen) following the manufacturer’s protocol. The remaining DNA was removed via DNase I (Invitrogen) treatment. Then, RNA was used as a template for cDNA synthesis using a High Capacity cDNA Reverse Transcription Kit (Applied Biosystems).

Real-time PCR reactions were carried out using a DyNAmo SYBR Green qPCR Kit (Thermo Scientific) on an Applied Biosystems StepOnePlus™ Real-Time PCR System. Reaction conditions were designed according to the kit manufacturer’s instructions. Primers were designed and their sequences were presented in Additional file 3: Table S1. Reactions were carried out in three replicates. The actin gene was used as a reference gene. The changes in transcript levels were presented as the relative quantification to the reference gene.

### Total DNA methylation analysis

Genomic DNA was isolated using DNeasy Plant Mini Kit (QUIAGEN) following the manufacturer’s protocol. The DNA integrity was examined by gel electrophoresis on 1.0% (w/v) agarose and the DNA amount was assessed by the spectrophotometry method.

The total DNA methylation was determined by the MethylFlash™ Methylated DNA Quantification Kit (Epigentek), which included a complete set of optimized buffers and reagents for colorimetric quantification of the global DNA methylation by specifically measuring levels of 5-methylcytosine (5-mC) in an ELISA-like microplate-based format (Varioscan Flash, Thermo Scientific).

### DNA methylation patterns of β-1,3-glucanase gene determination

In order to determine the pattern of DNA methylation, restriction enzymes MspI and HpaII (New England Biolabs) were used. Potential DNA methylation sites in the gene (CCGG islands) were determined by the program NEBcutter V2.0 from New England Biolab website. Using the Oligo 7 software, several primer pairs were designed and before the appropriate experiment they were checked in the gradient PCR program to estimate the correct annealing temperature.

DNA was digested by restriction enzymes overnight, and then it was used as a template for the PCR reaction. PCR products were electrophoresed on 1.0% agarose gels with ethidium bromide, and visualized under UV light. Levels of PCR products were measured by the densitometry analysis of the gel image using the Bio 1D program. The densitometry analysis was used to estimate patterns of DNA methylation. The pattern of promoter, exons and intron sites of methylation was calculated according to the equations:

$$ \mathrm{CCGG} = \mathrm{undigested}\ \mathrm{D}\mathrm{N}\mathrm{A}\ \hbox{--}\ \mathrm{D}\mathrm{N}\mathrm{A}\ \mathrm{digested}\ \mathrm{b}\mathrm{y}\ \mathrm{HpaII} $$

$$ \mathrm{CCmGG} = \mathrm{D}\mathrm{N}\mathrm{A}\ \mathrm{digested}\ \mathrm{b}\mathrm{y}\ \mathrm{HpaII}\ \hbox{--}\ \mathrm{D}\mathrm{N}\mathrm{A}\ \mathrm{digested}\ \mathrm{b}\mathrm{y}\ \mathrm{MspI} $$

$$ \mathrm{CmCmGG} = \mathrm{D}\mathrm{N}\mathrm{A}\ \mathrm{digested}\ \mathrm{b}\mathrm{y}\ \mathrm{MspI} $$

There were seven CCGG sites analysed (three in promoter (P) and exon (E) and one in intron(I)) in the β-1,3-glucanase gene. These sites were characterized by the following positions starting from ATG in DNA: P1 -1204-1201; P2 -1147-1144; P3 -344-341; E1 + 440-443; E2 + 1479-1482; E3 + 1763-1766; I1 + 2341-2344.

### Callose content

Determination of callose content in the flax plants was performed using a modified method described by Hirano [47]. A detailed description was presented in the previous paper [48]. 100 mg of fresh plant tissue was used for analysis.

### Isolation and fractionation of the cell wall polysaccharides

Isolation and fractionation of the cell wall components was performed using a modified version of the method described by Manganaris [49] and Vicente [50]. A detailed description was presented in the previous paper [48]. 100 mg of fresh plant tissue was used for analysis.

### Uronic acid measurement

The content of pectin was determined using the biphenyl method [51] after hydrolysis of the polysaccharides in sulphuric acid [52]. A detailed description was presented in the previous paper [48].

### Measurement of total sugars

The total sugar content in particular fractions was determined using the phenyl method after former hydrolysis of the polysaccharides in sulphuric acid. 0.6 ml of sulphuric acid was added to 0.3 ml of the supernatant after hydrolysis. The samples were shaken, 50 μl of 5% phenol in water was added, and then samples were incubated at 50°C for 20 min. After cooling, the amount of total sugars was measured with a spectrophotometer at 480 nm. Glucuronic acid was used for the calibration curve.

### Cellulose content

The cellulose content was determined using the colorimetric method with anthrone reagent, as described by Ververis [53]. A detailed description was presented in the previous paper [48]. 100 mg of fresh plant tissue was used for analysis.

### Lignin content

The determination of the total lignin content was performed using the modified acetyl bromide method [54]. A detailed description was presented in the previous paper [48]. 100 mg of fresh plant tissue was used for analysis.

### Polyamine extraction and analysis by UPLC

The isolation and fractionation of polyamines was performed using a modified version of the method described by Imai [55]. 100 mg of ground flax plants was extracted with 1 ml of 4% (w/v) perchloric acid (PCA) for 1 h at 4°C with shaking. After centrifugation, the pellet was resuspended in 0.5 ml of 4% PCA and 0.5 ml of 6 N HCl were added to 0.5 ml of the supernatant and both samples were hydrolysed at 110°C for 20 h. After drying in a speed-vac at 70°C, the residues were re-dissolved in 0.5 ml of 4% PCA. Subsequently, polyamine dansylation was performed: 0.2 ml aliquots were added to 0.2 ml of saturated sodium carbonate and 0.4 ml of dansyl chloride (5 mg/ml in acetone). After brief vortexing, the mixture was incubated in darkness at 60°C for 1 h. Excess dansyl reagent was removed by adding 0.2 ml of 150 mg/ml proline. Dansylated polyamines were extracted by 0.5 ml toluene, dried in a speed-vac concentrator and re-suspended in 0.2 ml of acetonitrile.

The samples were analysed using a Waters Acquity UPLC system with a 2996 PDA detector on an Acquity UPLC BEH C18, 2.1 × 50 mm, 1.7 μm column. The mobile phase was A = water and B = acetonitrile, in a gradient flow: 1 min at 60% A/40% B; to 2.5 min, gradient to 40% A/60% B; to 4 min, gradient to 25% A/75% B; to 5.5 min, gradient to 0% A/100% B; and to 6.5 min, gradient to 60% A/40% B with a 0.4 ml/min flow rate. The peak integration was done at 366 nm.

The results were standardized with mixtures of dansylated polyamine standards (putrescine, spermidine, spermine). Total polyamine content was determined as the sum of free polyamine, conjugated polyamine and polyamine bound to the cell wall.

### Infection tests

The determination of the plant resistance to the pathogen infection was performed on 4-week-old flax plants from F3 generation of B4 line grown in the tissue culture. The fungi were grown for 7 days at 18°C on potato–dextrose–agar (PDA) medium. The flax plants were transferred onto a solid culture of *Fusarium culmorum* and *Fusarium oxysporum*, and after 10–14 days, the numbers of infected flax plants (roots and hypocotyls) were determined and expressed as a percentage of the total flax plants used for the experiment. Approximately 18 flax plants (6 plants in each pot) for both *Fusarium* and control treatment were analysed. The experiment was repeated three times.

### Statistical analysis

All of the experiments were independently repeated at least three times. The results are presented as the averages of independent replicates ± standard deviations. Statistical analyses were performed using Statistica 7 software (StatSoft, USA). The significance of the differences between the means was determined using Student’s t test. P-values were given separately for each experiment (*P < 0.05, **P < 0.01).

## Additional files

Additional file 1: Figure S2. Level of genes expression involved in cell wall polymer metabolism and their targets in EMO-βGlu flax. EMO-βGlu flax (EB) and the control flax (Cf) were analysed with RT-PCR, actin was used as a reference gene. (A) Pectin synthesis: UDP-D-glucuronate-4-epimerase, GAE; α-1,4-galacturonosyltransferase 1, GAU1; α-1,4-galacturonosyltransferase 7, GAU7; rhamnogalacturonan II xylosyltransferase, RGXT; arabinosyltransferase, ARAD; pectin methyltransferases PMT (B) Pectin degradation: pectin methylesterases 1; PME1; pectin methylesterases 3, PME3; pectin methylesterases 5, PME5; polygalacturonase, PG; pectate lyase, PLL; pectin lyase, PL; (E) Hemicellulose synthesis: glucomannan 4-beta-mannosyltransferase 9-like, GMT; galactomannan galactosyltransferase, GGT; xyloglucan galactosyltransferase, XGT; (F) Hemicellulose degradation: endo-1,4-β-xylanase, XYN; 1,4-β-xylosidase, XYLb; 1,4-α-xylosidase, XYLa; α-galactosidases, GS; endo-β-mannosidase, MS; β-glycosidase, GLS; (I) Cellulose metabolism: cellulose synthases (CLS1-4), cellulase, Cel; (K) Lignin metabolism: phenylalanine/tyrosine ammonia-lyase, PAL; 4-Coumarate-CoA ligase, 4CL; naringenin-chalcone synthase, CHS; 4-coumaroyl-CoA:shikimate O-(hydroxycinnamoyl)transferase, HCT; p-coumarate 3-hydroxylase, C3H; caffeoyl-CoA O-methyltransferase, CCoAOMT; caffeate O-methyltransferase, COMT; sinapyl alcohol dehydrogenase, SAD; glucosyltransferase, GT (M) Polyamine synthesis: arginine decarboxylase, ADC; agmatine iminohydrolase, AIH; N-carbamoylputrescine amidase, NCPAH; arginase, ARG; ornithine decarboxylase, ODC; spermidine synthase, SPDS; spermine synthase, SPS; (N) Polyamine degradation: diamine oxidase, DAO; polyamine oxidase, PAO; (P) Callose synthesis: callose synthase, CALS; (C) and (G) The uronic acid contents in fractions of the cell wall; pectin fractions: WSF, CSF and NSF and hemicellulose fractions: K1SF and K4SF D and (H) Total sugar content in fractions of the cell wall; pectin fractions: WSF, CSF and NSF and hemicellulose fractions: K1SF and K4SF. (J) Cellulose content. (L) Lignin content. (O) Total polyamine content. Analyses of metabolic products were performed as described in the Methods section. Data represent the mean values ± SD from three independent experiments. The significance of the differences between the means was determined using Student’s t test (*P < 0.05, **P < 0.01). RQ – relative quantity. Additional file 2: Figure S1. Methylation of complementary sites of β-1,3-glucanase gene in flax treated with OLIGOs and in EMO-βGlu flax. The methylation of three sites in the promoter and one site in the exon of the β-1,3-glucanase gene in flax treated with OLIGOs and in EMO-βGlu flax. The analysis of flax treated with OLIGOs (B1, B2, B3 and B4) at 24 h and 48 h after exposure to OLIGOs in comparison with control, non-treated flax (C) and in EMO-βGlu flax (EB) in comparison with control flax from field (Cf) was determined by digesting genomic DNA with restriction enzymes HpaII-MspI with subsequent semi-quantitative PCR reaction, separation of the PCR product on agarose gel and quantification of the bands by densitometry. Data represent the mean ± standard deviations from three independent experiments. The significance of the differences between the means was determined using Student’s t test (*P < 0.05, **P < 0.01). Additional file 3: Table S1. Primer sequences for real-time RT-PCR reactions. Primer sequences designed for real-time PCR: (A) PR genes and actin. (B) DNA methylation genes. (C) Callose synthesis genes. (D) Pectin metabolism genes. (E) Hemicellulose metabolism genes. (F) Cellulose metabolism genes. (G) Lignin metabolism genes. (H) Polyamine metabolism genes.

## Abbreviations

EMO: Epigenetically modified organism
OLIGO: Oligonucleotide
