# Supplementary figures and images for: Oligonucleotide treatment causes flax β-glucanase up-regulation via changes in gene-body methylation
Source: BMC Plant Biol. 2014 Oct 5;14:261. doi: 10.1186/s12870-014-0261-z (PMC4209061; doi:10.1186/s12870-014-0261-z)

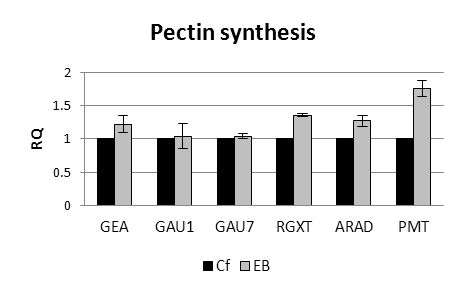

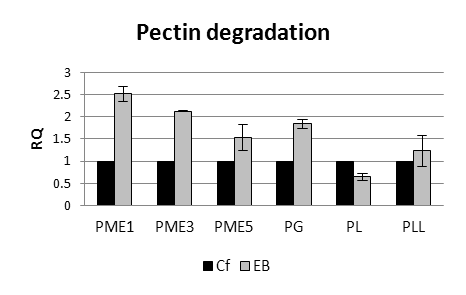

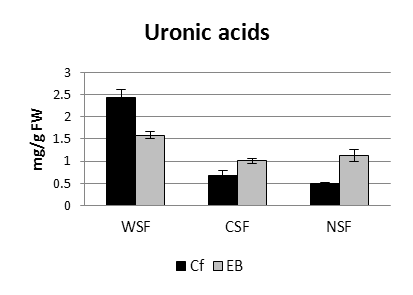

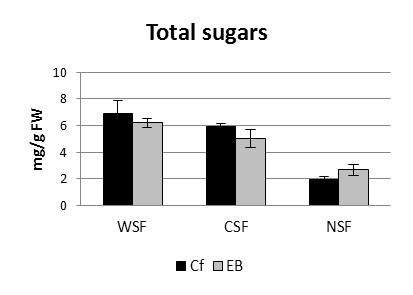


**D**

**A**

**C**

**B**

*

**

**

**

**

**

**

**

*

*

*

*


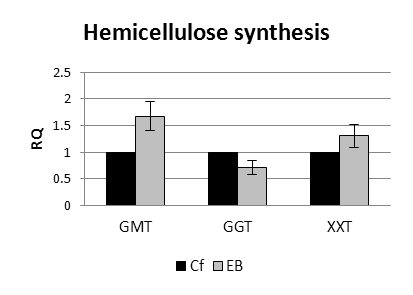

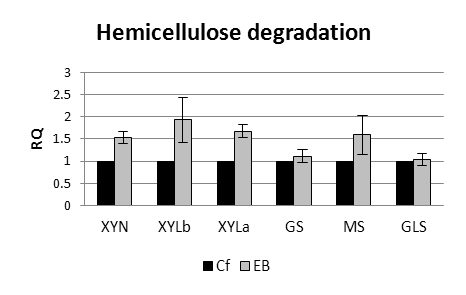

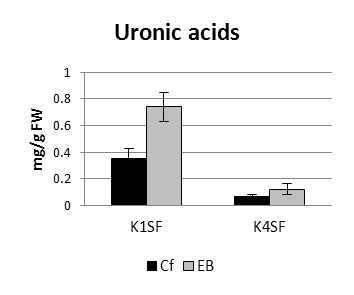

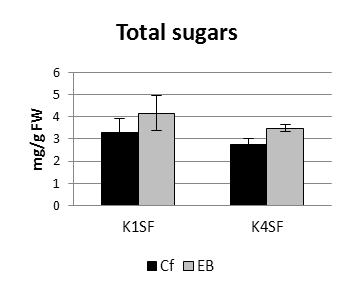


**G**

**E**

**

*

**

**

**

**

**F**

**H**


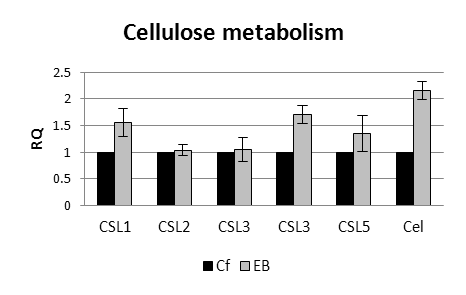

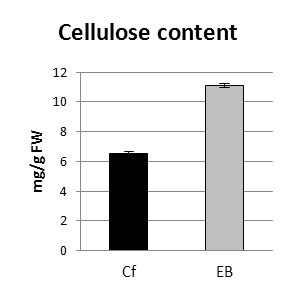


**I**

**J**

**

**

**

*


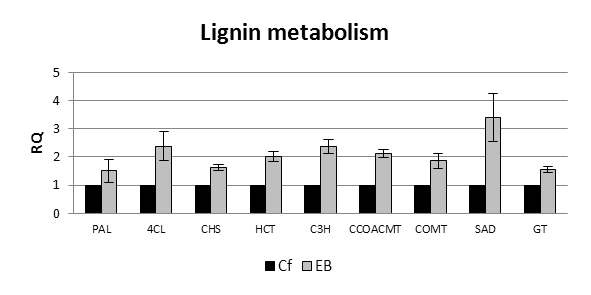

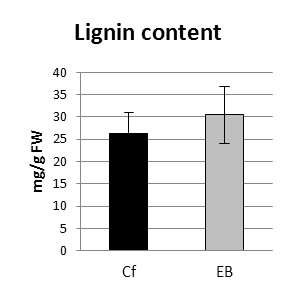


**L**

**K**

**

**

**

**

**

**

**

**


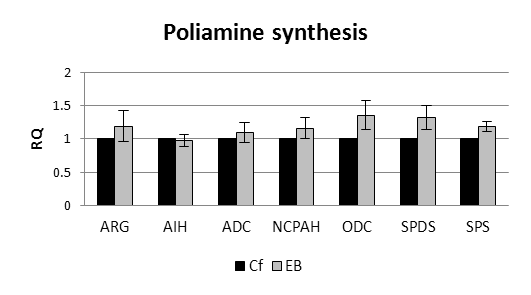

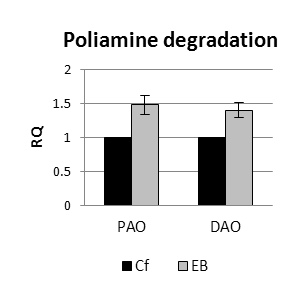

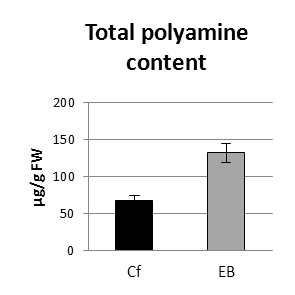

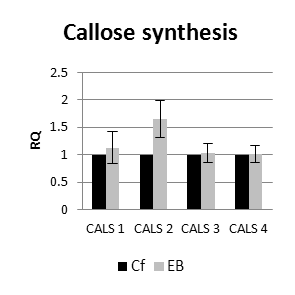


**O**

**N**

**M**

*

**

**

**

**P**

*

Supplement: Additional file 1: Figure S2. — Level of genes expression involved in cell wall polymer metabolism and their targets in EMO-βGlu flax. EMO-βGlu flax (EB) and the control flax (Cf) were analysed with RT-PCR, actin was used as a reference gene. (A) Pectin synthesis: UDP-D-glucuronate-4-epimerase, GAE; α-1,4-galacturonosyltransferase 1, GAU1; α-1,4-galacturonosyltransferase 7, GAU7; rhamnogalacturonan II xylosyltransferase, RGXT; arabinosyltransferase, ARAD; pectin methyltransferases PMT (B) Pectin degradation: pectin methylesterases 1; PME1; pectin methylesterases 3, PME3; pectin methylesterases 5, PME5; polygalacturonase, PG; pectate lyase, PLL; pectin lyase, PL; (E) Hemicellulose synthesis: glucomannan 4-beta-mannosyltransferase 9-like, GMT; galactomannan galactosyltransferase, GGT; xyloglucan galactosyltransferase, XGT; (F) Hemicellulose degradation: endo-1,4-β-xylanase, XYN; 1,4-β-xylosidase, XYLb; 1,4-α-xylosidase, XYLa; α-galactosidases, GS; endo-β-mannosidase, MS; β-glycosidase, GLS; (I) Cellulose metabolism: cellulose synthases (CLS1-4), cellulase, Cel; (K) Lignin metabolism: phenylalanine/tyrosine ammonia-lyase, PAL; 4-Coumarate-CoA ligase, 4CL; naringenin-chalcone synthase, CHS; 4-coumaroyl-CoA:shikimate O-(hydroxycinnamoyl)transferase, HCT; p-coumarate 3-hydroxylase, C3H; caffeoyl-CoA O-methyltransferase, CCoAOMT; caffeate O-methyltransferase, COMT; sinapyl alcohol dehydrogenase, SAD; glucosyltransferase, GT (M) Polyamine synthesis: arginine decarboxylase, ADC; agmatine iminohydrolase, AIH; N-carbamoylputrescine amidase, NCPAH; arginase, ARG; ornithine decarboxylase, ODC; spermidine synthase, SPDS; spermine synthase, SPS; (N) Polyamine degradation: diamine oxidase, DAO; polyamine oxidase, PAO; (P) Callose synthesis: callose synthase, CALS; (C) and (G) The uronic acid contents in fractions of the cell wall; pectin fractions: WSF, CSF and NSF and hemicellulose fractions: K1SF and K4SF D and (H) Total sugar content in fractions of the cell wall; pectin fractions: WSF, CSF and NSF and hemicel [file 12870_2014_261_MOESM1_ESM.doc]
