# Supplementary material for: Oligonucleotide treatment causes flax β-glucanase up-regulation via changes in gene-body methylation
Source: BMC Plant Biol. 2014 Oct 5;14:261. doi: 10.1186/s12870-014-0261-z (PMC4209061; doi:10.1186/s12870-014-0261-z)
Supplement: Additional file 2: Figure S1. — Methylation of complementary sites of β-1,3-glucanase gene in flax treated with OLIGOs and in EMO-βGlu flax. The methylation of three sites in the promoter and one site in the exon of the β-1,3-glucanase gene in flax treated with OLIGOs and in EMO-βGlu flax. The analysis of flax treated with OLIGOs (B1, B2, B3 and B4) at 24 h and 48 h after exposure to OLIGOs in comparison with control, non-treated flax (C) and in EMO-βGlu flax (EB) in comparison with control flax from field (Cf) was determined by digesting genomic DNA with restriction enzymes HpaII-MspI with subsequent semi-quantitative PCR reaction, separation of the PCR product on agarose gel and quantification of the bands by densitometry. Data represent the mean ± standard deviations from three independent experiments. The significance of the differences between the means was determined using Student’s t test (*P < 0.05, **P < 0.01). [file 12870_2014_261_MOESM2_ESM.doc]

Additional file 2: Figure S1


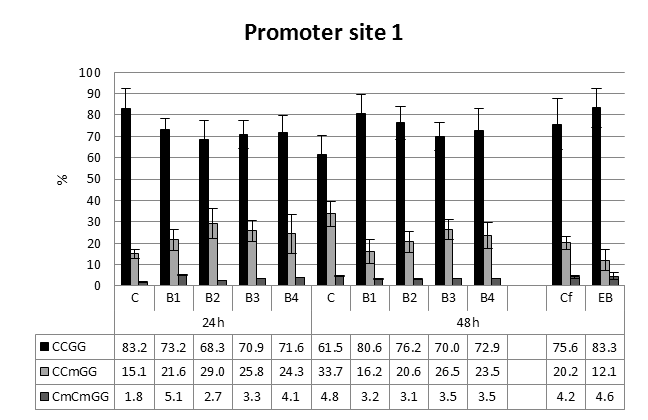


OLIGO greenhouse


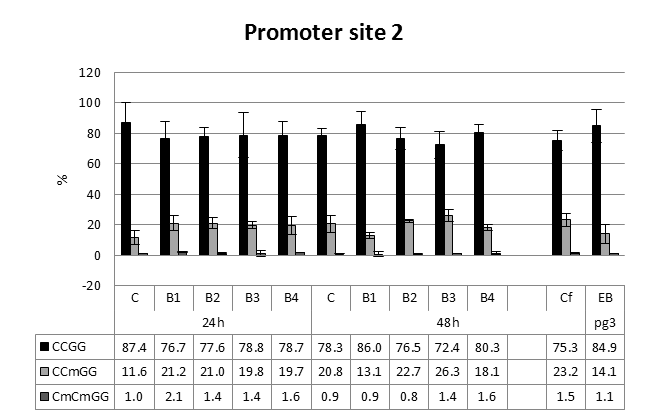


OLIGO greenhouse


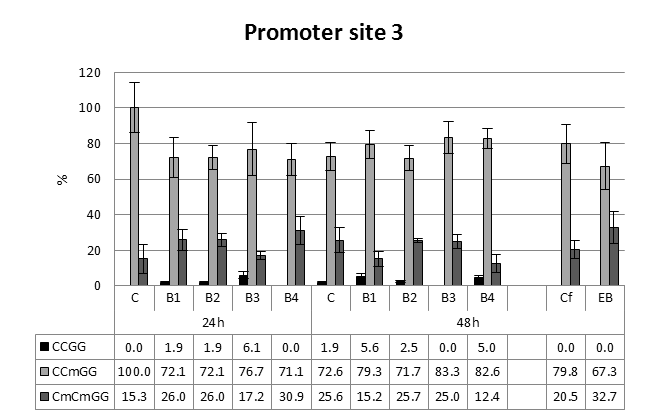


OLIGO greenhouse


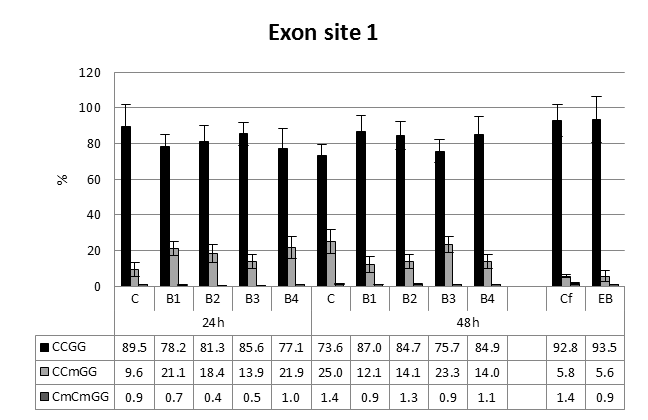


OLIGO greenhouse
