# Supplementary material for: Oligonucleotide treatment causes flax β-glucanase up-regulation via changes in gene-body methylation
Source: BMC Plant Biol. 2014 Oct 5;14:261. doi: 10.1186/s12870-014-0261-z (PMC4209061; doi:10.1186/s12870-014-0261-z)
Supplement: Additional file 3: Table S1. — Primer sequences for real-time RT-PCR reactions. Primer sequences designed for real-time PCR: (A) PR genes and actin. (B) DNA methylation genes. (C) Callose synthesis genes. (D) Pectin metabolism genes. (E) Hemicellulose metabolism genes. (F) Cellulose metabolism genes. (G) Lignin metabolism genes. (H) Polyamine metabolism genes. [file 12870_2014_261_MOESM3_ESM.doc]

Additional file 3: Table S1

1. PR GENES and ACTIN

| **GENE** | **FORWARD PRIMER** | **REVERSE PRIMER** |
| --- | --- | --- |
| β-1,3-GLU1 | CTAGGCAGCGTGAAAGC | CGTCGAAGAGGTTGGTG |
| β-1,3-GLU2 | GATCTGGTCAAAGAGGTTGGTATAA | GTCCTTCTTCTTCCTCGATG |
| CHIT | CATCCAATGAATGGCCTT | GGCTGTTCGGAATGATATCTC |
| PME1 | GAGCTGGAACCACATGC | AGCCTTCACGTATATTACGC |
| ACT | CCGGTGTTATGGTTGGAAT | TGTAGAAAGTGTGATGCCAAA |

1. DNA METHYLATION

| **GENE** | **FORWARD PRIMER** | **REVERSE PRIMER** |
| --- | --- | --- |
| CMT1 | CAGATT TCGCTC CACAGT A | AGAAAT GTCCCA TTGCTC TAT |
| CMT3 | AAAGGG TGCTAA CTTCAG G | GACCAA ATGGTT TAGACG ATGT |
| DME | ATGGCT ACGGAG GCTACT TA | TGTTTC ACCTGG TGTCCA TA |
| ROS1 | GCACTG AGAAGA AGTGCC | CTTAAT GCGTGC TGCAAG |
| AGO1 | CTGGTA GTTCGG CTCCAA | AGGAGC TGCTCA TTTCTT |
| AGO4 | ATGGAC GAGGTT GGTTT | CTTAAT GAATTG TCCCAT TTGACT |
| RDR2 | AAGTTA TTCCTG ACATAG AGGTG | AACGCC TTTATA CCCGC |

1. CALLOSE SYNTHESIS

| **GENE** | **FORWARD PRIMER** | **REVERSE PRIMER** |
| --- | --- | --- |
| CalS1 | GCTTCCATGTGGTTCTTAGT | CTCCCAGCTCTTATTCGC |
| CalS2 | CCTCTTCCATGTGGTTCTTAG | TTATTTGCAGGCACACCG |
| CalS3 | AGAGCAAGATAAAGACGACC | TATTGAGCAGAGATGTTTACAGG |
| CalS4 | CTTTACTTCACGGAGGTGC | GCCCGAAGATTTCATAGACGA |

1. PECTIN METABOLISM

| **GENE** | **FORWARD PRIMER** | **REVERSE PRIMER** |
| --- | --- | --- |
| GAE | CCGAGGTACTCTTTCCTGA | CATACTTCTCGTTTACGAGGAAT |
| GAU 1 | AATTTAAGTGGCTTAATTCATCCTAC | GTGATTGAGCATTGAAAGATACTTG |
| GAU 7 | TGCAATATCCAAGGCACAGTTA | AGACAATGATGGCTCTTAGG |
| ARAD | ATTAACCAAGTATCCCGGC | TACTCCGTCAGCATGCAG |
| RGXT | TCCTGCGCTTGATTCACA | GGATCTCCCAGCCAAAC |
| PMT | ACGATTCTTGATATAGGCTGT | AGAACCAATCATCGCCG |
| PME1 | GAGCTGGAACCACATGC | AGCCTTCACGTATATTACGC |
| PME3 | AGTGGGATGGCAACTTT | TGAACCGACCGGGAGTA |
| PME5 | CGTAGTGGGTGACAGATTTAT | GAGGGAGTGGACGTAGAG |
| PG | GGATCAAGACTGCTGTGG | CAACAGGGATCGCGTTAG |
| PLL | GAGCATGTGATCGTATGCAA | CCTGGAAATGGTAATGTCAGT |
| PL | AACTACGAGAGAATCAAAGGAAC | GGGAGTATTCAACTCTCCG |

1. HEMICELLULOSE METABOLISM

| **GENE** | **FORWARD PRIMER** | **REVERSE PRIMER** |
| --- | --- | --- |
| GMT | GCTAGAGTGCCAAAGGTG | GACCGATGTCCGAGTTATG |
| GGT | GTGTTTCTGATTCGTAACTGC | GAGTAAGTAGATCAACGCCG |
| XXT | AGATCGATTATTGCAGGCT | GCATCACTGTCCATCCAC |
| XYN | ACAGCAAGGAAGTCTTAACTAC | TCACAGCTTTCATTAAGTCATCT |
| XYL b | AAAGAACACCAGGATCTAGC | CTGCATAGTTTCCTAGAAGAGTC |
| XYL a | CAAGTATGATGATCAGCCTTTC | ATCTCGTTCATGTCGATCC |
| GS | CAGTCTCCTGGCGTGTTA | CAACTTTGACTCCATCTATGC |
| MS | TGACTATCTCGATGGCACAC | GGCATTCCTACAGAGCCAA |
| GLS | GATAAGGCCTCCGGATAAC | ATGAATACAGTGAATCGGGAC |

1. CELLULOSE METABOLISM

| **GENE** | **FORWARD PRIMER** | **REVERSE PRIMER** |
| --- | --- | --- |
| CSL1 | GGTCGAAATCTTCTTCTCACG | CGAGAATAAAGAGAGTGCGG |
| CSL2 | CTTACTCGCCAGTCCAAG | GTCACGTTGAGAGTTGACAC |
| CSL3 | AGTAGTAGGCTCAAGTTCCGA | GAATGCTATGTTTAGAGACTGGAC |
| CSL4 | TAATGTTGGCATCTACCCTTTC | CATTTGATCTCTAGGACGGC |
| CSL5 | TCAATGTCGGCATCTACCC | TGGCTAGACCAATGAGGC |
| CEL1 | CCATTACCCAAAGCACGTC | CTCGGTGTAGTTGTAGTTCATTC |

1. LIGNIN METABOLISM

| **GENE** | **FORWARD PRIMER** | **REVERSE PRIMER** |
| --- | --- | --- |
| PAL | GTTCTG TTTGAA GCCAAT GT | TGTAAG CACTCC CGTCG |
| 4CL | GCAGAAATGAAGATCGTCG | GTATGTAACCACCCTTGCT |
| CHS | AAATGG GGAGAA TGGAAG GA | CGCACG ATTCAA ATAGTG AGA |
| HCT | GTCGATATTCAAGCTGACCC | GTGGCGATGTACAGTTTAGT |
| C3H | CCATTGGAGTTCAAACCAGA | AGCAAGTGTCCCAACATAG |
| CCOACMT | CGGACAAGGACAACTACAT | ACGAAGTCCCTGTAGTACCTAA |
| COMT | CTCTTGGCTTCTTACTCTGTT | TGAGGACTTTGTCCTGGTT |
| SAD | CTACCTACGGAGGCTACT | GCTTGTCTAGCCCATAGAAC |
| GT | ATGCAGTGTGCATTCCAT | CGAATCGGAACGAAGGTAG |

1. POLYAMINE METABOLISM

| **GENE** | **FORWARD PRIMER** | **REVERSE PRIMER** |
| --- | --- | --- |
| ADC | GAGGAGCTTGATTTGGTGA | CACGCTGAGAATCTGAGTT |
| AIH | GTCTACTGCAACTGATGCTAA | AGAAGCAGCAAGTCTTGT |
| NCPAH | AAGCACAAAGGGAGGAC | CTATGGAATTGTAATGAGCATTGTT |
| ARG | CTGATGTTGGTGATGTCCC | CCTCCAAGTTTCTCAGATACAG |
| ODC | CTGGATGAGTGTTTCTCAGG | GCAATTCATCGACCCGTA |
| SPDS | AAAGCAGTCCTGGTAGTTG | CGTGGATCTTCGAATCCG |
| SPS | GGCTATTCCTTCCTGTTGTC | GGCCACATAGGATTGTTAAAGTA |
| DAO | CAAAGTCGGCATGATCG | GGGTTTACGTTGACCAGAG |
| PAO | ATGGGAGGACGTTTGTAG | GCCAGAACACATTGTCGAA |
